# Guided Implant Placement for Precise and Efficient Dental Implant Positioning: A Case Report

**DOI:** 10.7759/cureus.109517

**Published:** 2026-05-23

**Authors:** Richard M Cavero, Ana C Gramcko, Luis Fernandez Cotera, Mayree Ferreira-Alfonzo, Alexo A Faria

**Affiliations:** 1 Dentistry, Metro Dental Associates, Morristown, USA; 2 Dentistry, Lake Nona Dental, Orlando, USA; 3 Dentistry, Private Practice, Houston, USA; 4 Dentistry, Private Practice, Miami, USA; 5 Dentistry, North Tampa Family Dental, Tampa, USA

**Keywords:** dental implant, guided implant placement, guided surgery, implant accuracy, implant angulation, implant positioning, mandibular molar, osteotomy preparation, prosthetic planning, surgical guide

## Abstract

Guided implant surgery has become an important approach to improve the accuracy and predictability of dental implant placement by providing control over angulation, depth, and positioning. This case report describes the use of a laboratory-fabricated surgical guide for implant placement in a 42-year-old male patient presenting with a missing mandibular molar. Clinical and radiographic evaluations were performed to assess the edentulous site and to plan implant positioning. A conventional impression was obtained to fabricate the surgical guide, which was designed to direct the osteotomy and maintain an accurate implant trajectory. During the procedure, the guide was adapted intraorally to ensure stability, and osteotomy preparation was performed through the guide to achieve controlled drilling along the planned path. The implant was placed using the guide to maintain proper angulation and positioning. Postoperative radiographic evaluation confirmed appropriate implant alignment consistent with the intended trajectory. The use of a surgical guide facilitated a predictable surgical workflow, reduced the risk of deviation, and contributed to a favorable clinical outcome. This case highlights a practical and effective technique for achieving accurate implant placement in routine clinical practice.

## Introduction

Accurate implant placement is essential for achieving optimal functional and prosthetic outcomes in implant dentistry. Deviations in angulation, depth, or position can compromise biomechanics, esthetics, and long-term success [1,2]. For this reason, various techniques have been developed to improve the precision and predictability of implant placement [3,4].

Tooth-supported static surgical guides have become an important tool in implant dentistry, allowing clinicians to transfer a planned implant position into the clinical setting with greater accuracy. In this case, a conventional, laboratory-fabricated tooth-supported acrylic guide with metal drill sleeves was used, representing a fully guided protocol directing both osteotomy preparation and implant insertion. Accuracy was ensured through planning based on a diagnostic wax-up, cone-beam computed tomography (CBCT) imaging, and a conventional cast impression, enabling precise transfer of the planned implant trajectory to the surgical site. By controlling the direction of osteotomy preparation, these guides help maintain proper angulation and reduce the risk of positioning errors, particularly in posterior regions where access and visibility may be limited [5,6].

Although digital workflows and computer-guided systems are increasingly used, conventional laboratory-fabricated surgical guides remain a practical and accessible option in routine clinical practice [7-9]. These guides can be produced using standard impression techniques and are effective for achieving precise implant positioning without the need for advanced digital equipment.

However, few reports provide step-by-step technical details and objective criteria for intraoperative verification of guide seating and stabilization, and for postoperative radiographic confirmation. This case report addresses this gap by demonstrating a practical workflow, including guide adaptation, fully guided osteotomy and implant insertion, and objective postoperative evaluation, providing clinicians with a reproducible method to enhance accuracy and interpretability of clinical outcomes.

## Case presentation

A 42-year-old male patient presented with a missing mandibular right first molar (Universal #30; Fédération Dentaire Internationale {FDI} 46) seeking implant rehabilitation. The patient reported no significant medical history and was in good general health. Clinical examination revealed adequate keratinized tissue. The edentulous site measured approximately 8 mm mesio-distally and 7 mm bucco-lingually, with sufficient bone height for implant placement. Preoperative assessment was performed using periapical radiographs, which allowed evaluation of alveolar ridge dimensions and proximity to adjacent teeth and the inferior alveolar nerve (IAN). The planned implant was a Straumann bone level tapered (BLT) 4.1 × 10 mm (Basel, Switzerland: Straumann AG), with an insertion torque of 35 Ncm and an initial stability measured by 72 Implant Stability Quotient (ISQ), selected based on these measurements. The treatment plan included a conventional two-stage healing protocol with delayed loading.

The treatment plan consisted of implant placement using a laboratory-fabricated, tooth-supported static surgical guide to improve accuracy and control during the procedure. A conventional polyvinyl siloxane (PVS) impression of the mandibular arch was obtained and poured with high-strength dental stone (Virtual Putty Regular Set; Schaan, Liechtenstein: Ivoclar Vivadent) to produce an accurate cast. A bite registration was also obtained to maintain proper occlusion. A diagnostic wax-up was performed to determine the ideal prosthetic emergence and planned implant position. The laboratory then fabricated the guide from clear photopolymer resin (NextDent SG Resin, 3 mm thickness; Soesterberg, Netherlands: 3D Systems), incorporating metal drill sleeves (5 mm diameter × 5 mm height) to transfer the planned implant trajectory from the wax-up/cast to the surgical site. The guide was compatible with the Straumann BLT implant system and the Straumann guided surgery kit (Basel, Switzerland: Straumann AG), enabling fully guided osteotomy and implant insertion, with the implant inserted directly through the guide sleeves. While a conventional impression was used in this case, digital impressions can also be integrated into guided workflows as an alternative method for planning and guiding fabrication.

**Figure 1:**
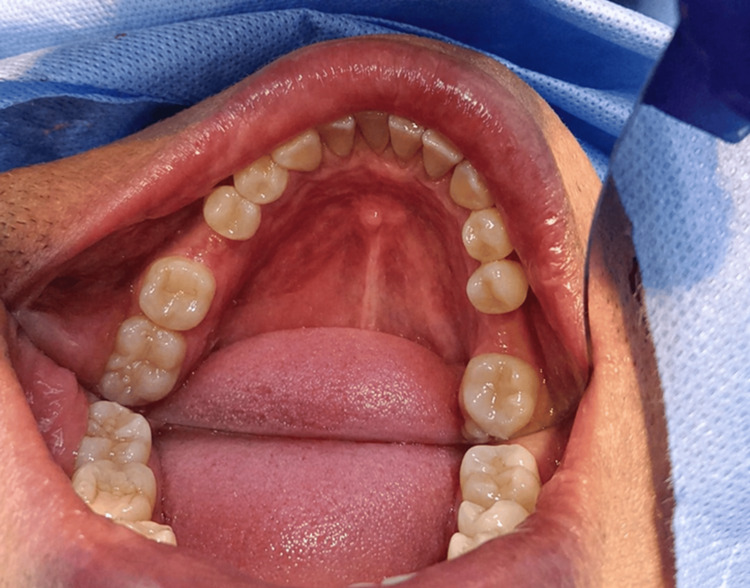
Preoperative intraoral occlusal view showing edentulous site at tooth #30 planned for implant placement. Intraoral occlusal view of the mandibular arch showing the edentulous site at tooth #30 (Universal #30; FDI 46). The adjacent teeth are present and intact, and the keratinized gingival tissue around the edentulous site is visible.

At the surgical appointment, the guide was evaluated intraorally for proper fit and stability. Seating was verified through visual inspection, ensuring full contact with occlusal stops and confirming that there was no rocking or movement. Fit-check material (silicone occlusal paste) was applied between the guide and teeth to detect any discrepancies. Fixation pins were not required, as adequate stability was achieved with the existing dentition and guide design. Once seating was confirmed, the guide was positioned over the edentulous site at the mandibular right first molar (Universal #30; FDI 46) (Figure 1).

**Figure 2:**
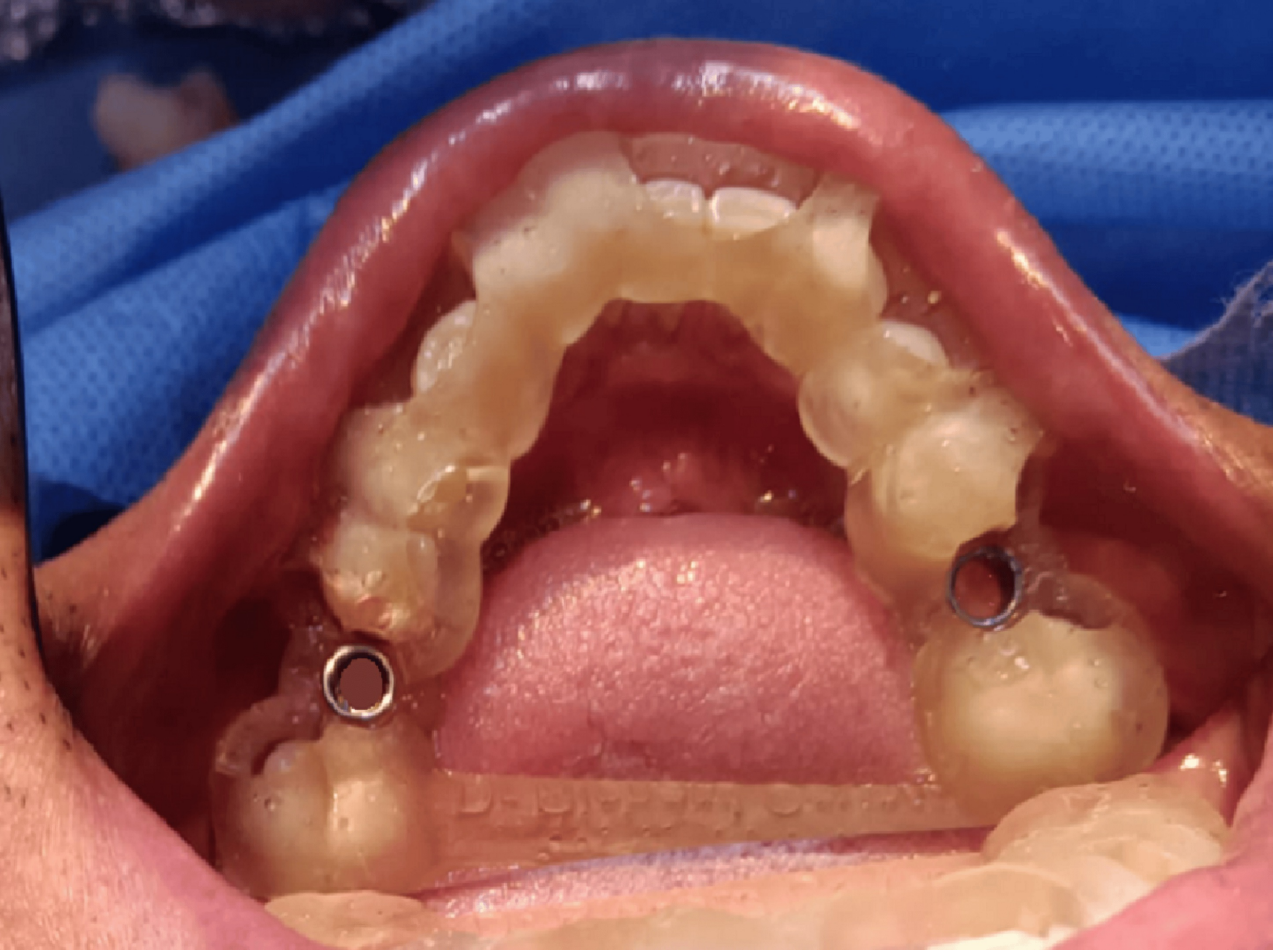
Intraoral view showing surgical guide positioned over the mandibular arch, demonstrating guide stability and alignment at the edentulous site of tooth #30. Intraoral view of the mandibular arch with the surgical guide seated over the edentulous site at tooth #30 (Universal #30; FDI 46). The guide is adapted to the adjacent teeth and incorporates metal drill sleeves indicating the planned implant trajectory.

**Figure 3:**
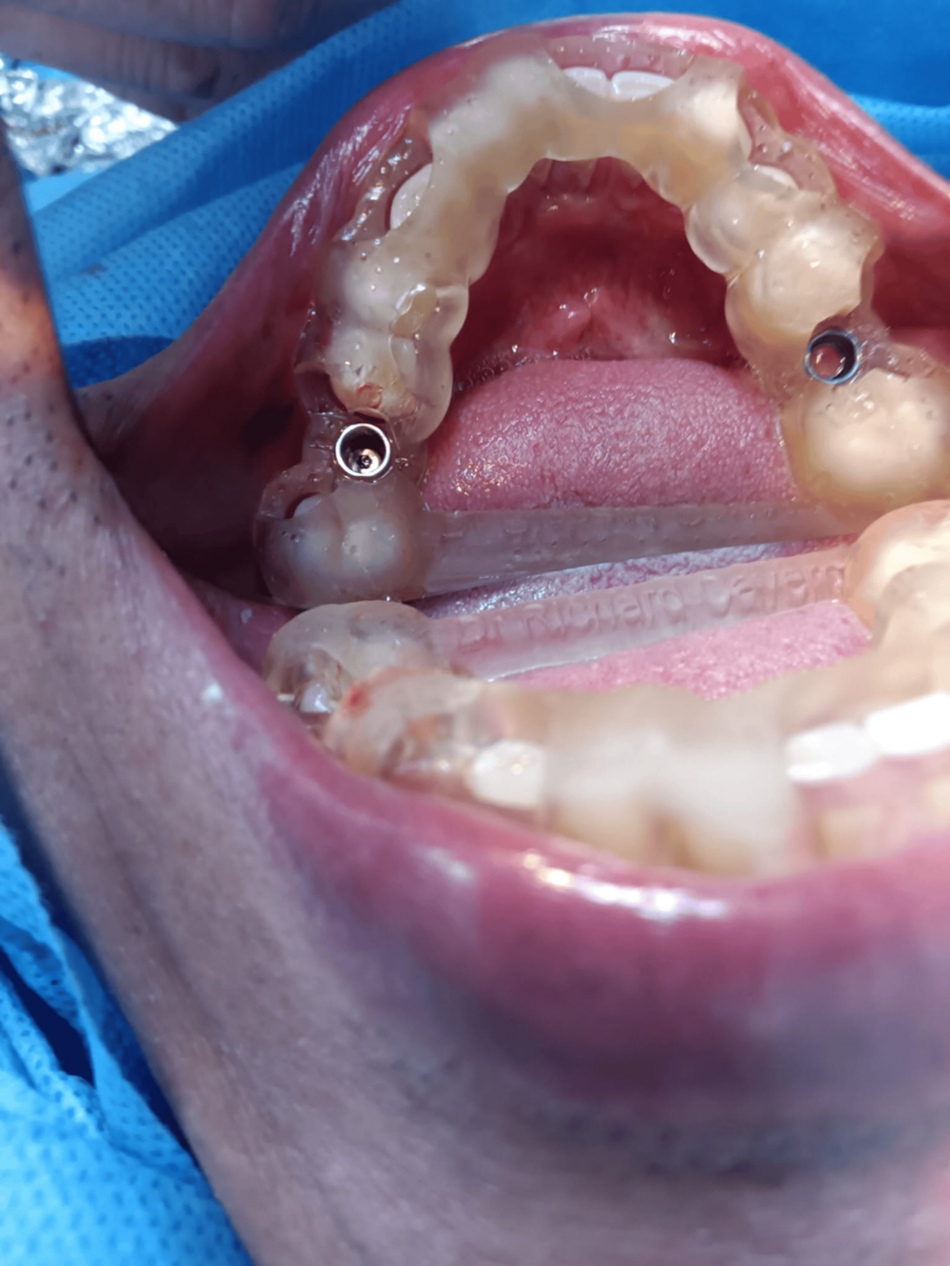
Intraoral view showing implant placement at tooth #30 using a surgical guide. Intraoral view of the mandibular right first molar site (Universal #30; FDI 46) after implant placement. The implant is positioned within the edentulous site, with adjacent teeth visible and soft tissue intact.

Osteotomy preparation followed a fully guided drill sequence using the Straumann guided surgery kit; pilot, intermediate, and final-diameter drills were used sequentially. Depth control was ensured by the metal drill sleeves, which acted as built-in stops for all drills, controlling both angulation and depth. Copious sterile saline irrigation was applied continuously to prevent heat generation and protect the bone during drilling. Implant insertion was performed through the guide sleeve, ensuring full control of angulation and depth and avoiding freehand placement (Figures 2, 3).

**Figure 4:**
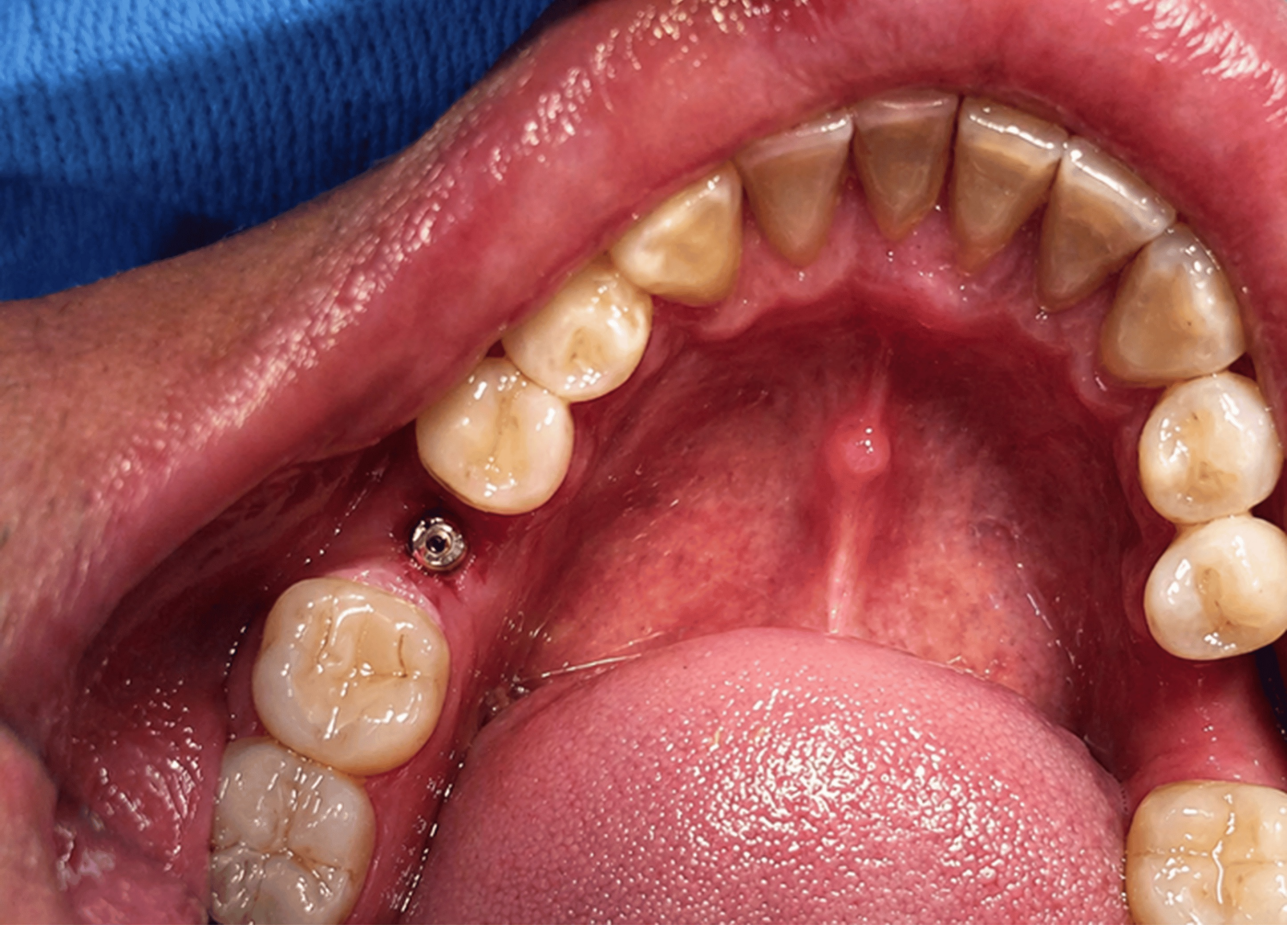
Intraoral view showing the implant placed at tooth #30. Intraoral view of the mandibular right first molar site (Universal #30; FDI 46) following implant placement. The implant is positioned within the edentulous site, with adjacent teeth and surrounding soft tissue visible.

**Figure 5:**
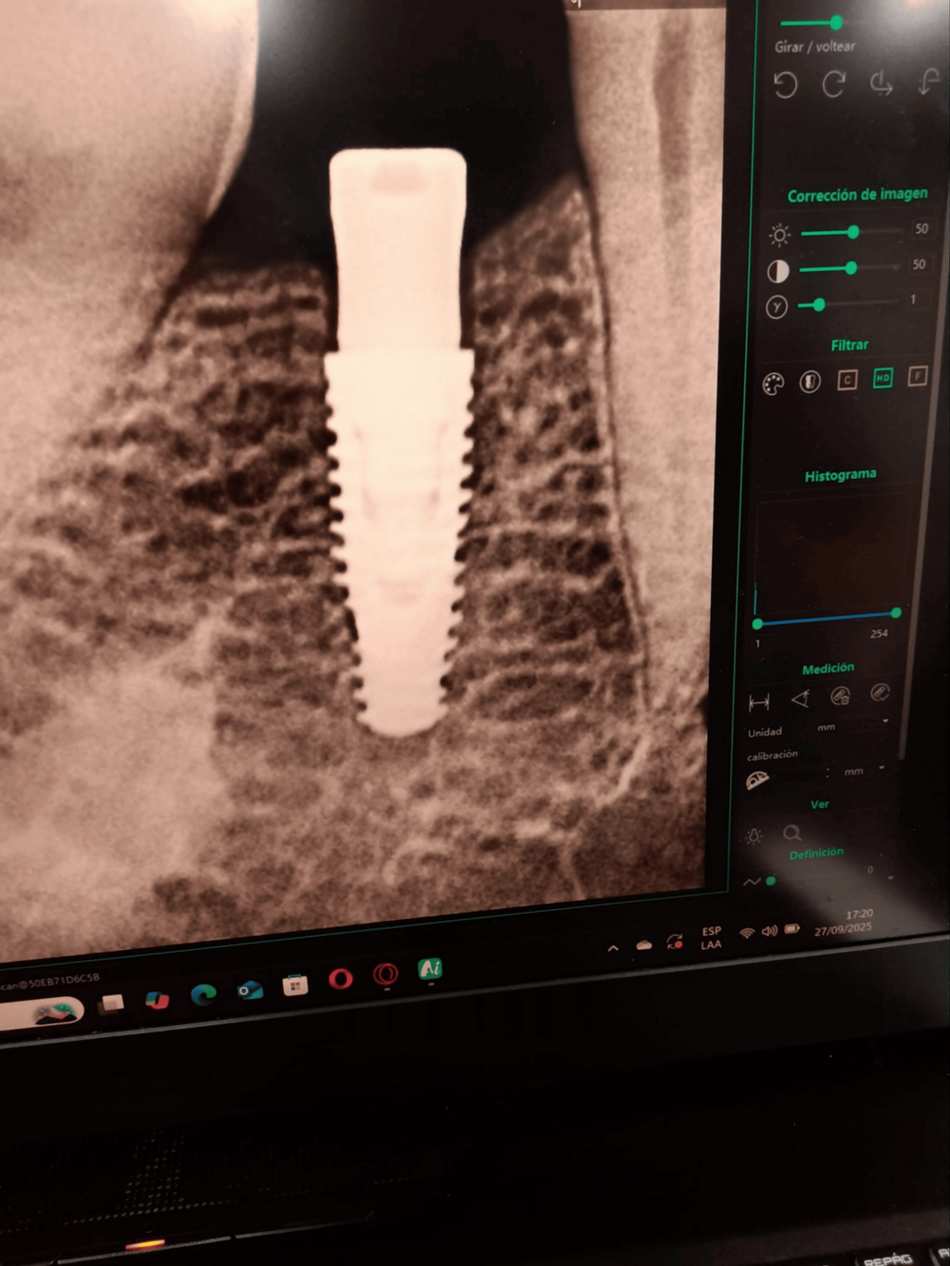
Postoperative radiograph showing implant placement at tooth #30. Periapical radiograph of the mandibular right first molar site (Universal #30; FDI 46) after implant placement. The implant fixture is visible within the alveolar bone, with adjacent teeth and surrounding bone structures observable.

Following completion, the guide was removed, and the implant position was clinically assessed (Figure 4). Postoperative evaluation was performed using periapical radiographs. Implant alignment was assessed based on axial angulation, mesio-distal position relative to adjacent teeth, and vertical depth of the implant platform. Placement was considered accurate if deviations were ≤2° in angulation, ≤1 mm mesio-distally, and ≤1 mm vertically compared to the preoperative plan. Clinically meaningful deviations could compromise restorative emergence or risk proximity to adjacent roots or the IAN. Postoperative measurements confirmed that the implant met these criteria (Figure 5).

Postoperative follow-up

The patient was evaluated at one week, two weeks, and three months postoperatively. At the one-week and two-week visits, the patient reported no pain or swelling, and soft tissues around the implant site appeared healthy with no signs of infection or inflammation. At three months, before restoration delivery, clinical examination confirmed stable keratinized tissue, absence of mobility, and proper soft tissue healing. Standardized periapical radiographs at three months demonstrated maintained crestal bone levels with no radiographic signs of peri-implant bone loss, confirming stability of the implant and appropriate osseointegration. The implant was subsequently restored following the planned delayed loading protocol.

The procedure was completed without complications. The use of the fully guided static surgical guide facilitated a controlled surgical workflow, and in this case, the implant position was consistent with the intended trajectory based on postoperative imaging. This technique may improve accuracy, but these observations are limited to a single case.

## Discussion

Accurate implant positioning is a critical factor for achieving long-term functional and prosthetic success in implant dentistry. Deviations in angulation, depth, or position can negatively affect load distribution, prosthetic alignment, and overall treatment outcomes. The use of surgical guides has been shown to improve precision by transferring the planned implant position to the clinical setting in a controlled manner [1,2].

In this case, a laboratory-fabricated, tooth-supported static surgical guide was used to perform a fully guided protocol, directing both osteotomy preparation and implant insertion. This modality provided a standardized trajectory reference, helping to control implant angulation, depth, and positioning during the procedure. This is particularly relevant in posterior regions, such as the mandibular first molar area, where limited visibility and access can increase the risk of deviation [3,4].

While a single case cannot definitively demonstrate superiority, fully guided, tooth-supported static guides may help reduce operator-dependent variability compared to freehand placement. Prior studies evaluating conventional, laboratory-fabricated, tooth-supported guides have reported mean angular deviations of 2°-3° at the entry point and 2°-4° at the apex, with linear deviations at the platform and apex typically ≤1.5 mm, supporting the accuracy and reproducibility of this approach in workflows similar to the one used here [5,6].

An additional benefit observed in this case was the facilitation of a controlled surgical workflow. The guide required only a single seating verification, with no rocking, repositioning, or intraoperative angulation corrections, illustrating practical efficiency. Postoperative evaluation was performed using standardized periapical radiographs with the paralleling technique. Implant alignment was assessed objectively using angular deviation (≤2°), coronal/platform deviation (≤1 mm), apical deviation (≤1 mm), and vertical depth deviation (≤1 mm) relative to the preoperative plan. Clinically meaningful deviations could compromise restorative emergence or risk proximity to adjacent roots or the inferior alveolar nerve, highlighting the importance of guided protocols in minimizing such risks [7,8]. Objective metrics from this case include the use of a Straumann BLT 4.1 × 10 mm implant, insertion torque of 35 Ncm, and ISQ of 72, confirming adequate primary stability and proper prosthetic planning. Recent literature further demonstrates improved accuracy and favorable clinical outcomes specifically for tooth-supported static, laboratory-fabricated guides, supporting the relevance of this approach to the reported case [9].

Despite these advantages, surgical guides are dependent on accurate fabrication and proper intraoral adaptation. Any discrepancy in fit may compromise the final implant position, emphasizing the importance of careful verification of guide seating prior to osteotomy preparation. Limitations of this report include the absence of long-term follow-up, CBCT-based quantitative deviation analysis, and explicit safety distance measurements to the inferior alveolar nerve. Therefore, while this case demonstrates favorable positioning, objective alignment, and workflow efficiency, the findings are limited to a single case and should not be generalized.

## Conclusions

Within the limitations of a single case report, the use of a laboratory-fabricated, tooth-supported static surgical guide helped standardize osteotomy trajectory and implant insertion at site #30. In this case, the guide was associated with an uneventful immediate postoperative course, with controlled angulation, implant depth, and alignment as assessed by standardized periapical radiographs.

Because implant dimensions, stability metrics, and long-term follow-up outcomes were not quantified, further case series or controlled studies with CBCT-based planning and deviation analysis are needed to confirm the accuracy benefits and long-term prosthetic and peri-implant outcomes. Conventional laboratory-fabricated guides remain accessible options that may help standardize implant trajectory in selected cases; however, these findings cannot be generalized, and additional studies are required to evaluate reproducibility and clinical efficacy.
